# Quantitative and qualitative analysis of pulmonary arterial hypertension fibrosis using wide-field second harmonic generation microscopy

**DOI:** 10.1038/s41598-022-11473-5

**Published:** 2022-05-05

**Authors:** Yaraslau Padrez, Lena Golubewa, Tatsiana Kulahava, Tatyana Vladimirskaja, Galina Semenkova, Igor Adzerikho, Olga Yatsevich, Nadezda Amaegberi, Renata Karpicz, Yuri Svirko, Polina Kuzhir, Danielis Rutkauskas

**Affiliations:** 1 Center for Physical Sciences and Technology, Vilnius, Lithuania; 2 Institute for Nuclear Problems of Belarusian State University, Minsk, Belarus; 3 Central Scientific and Research Laboratory of BelMAPE, Minsk region, Belarus; 4 Belarusian State University, Minsk, Belarus; 5 State Educational Establishment BelMAPE, Minsk, Belarus; 6 Department of Physics and Mathematics, University of Eastern Finland, Institute of Photonics, Joensuu, Finland

**Keywords:** Biophysics, Diseases, Medical research, Signs and symptoms, Optics and photonics

## Abstract

We demonstrated that wide-field second harmonic generation (SHG) microscopy of lung tissue in combination with quantitative analysis of SHG images is a powerful tool for fast and label-free visualization of the fibrosis pathogenesis in pulmonary arterial hypertension (PAH). Statistical analysis of the SHG images revealed changes of the collagen content and morphology in the lung tissue during the monocrotaline-induced PAH progression in rats. First order statistics disclosed the dependence of the collagen overproduction on time, the second order statistics indicated tightening of collagen fiber network around blood vessels and their spreading into the alveolar region. Fourier analysis revealed that enhancement of the fiber orientation in the collagen network with PAH progression was followed with its subsequent reduction at the terminating phase of the disease. Proposed approach has potential for assessing pulmonary fibrosis in interstitial lung disease, after lung(s) transplantation, cancer, etc.

## Introduction

Pulmonary arterial hypertension (PAH) is a clinical syndrome with a bleak prognosis^1^. There is a common belief that PAH is associated with the dysfunction or damage of the endothelium, leading to the remodeling of the pulmonary vessels, an increase in vascular resistance and blood pressure in the pulmonary artery^2^. In adventitia, the outermost layer of the blood vessel wall, there is an increased production of extracellular matrix (ECM) components, including collagen, elastin, fibronectin, and tenascin. Remodeling of large arterial vessels is expressed in fewer elastin fibers, increased collagen production and calcium deposition^3^, and is often accompanied by a violation of collagen degradation^4^. This imbalance causes the progression of interstitial and perivascular reactive fibrosis, which is manifested itself in massive ECM deposition^5^, scarring^6^, proximal pulmonary artery stiffening^7^, increased pulmonary vascular resistance^8^. These conditions are life-threatening and may lead to right ventricular failure and death if untreated.

Remodeling of ECM plays a central role in the pathogenesis of the PAH, making it an attractive novel therapeutic target^3^. The collagen morphology (e.g. organization of collagen fibers in bundles and whorls as well as their density, thickness and orientation) contains a valuable information on the normal/pathological conditions in tissue^9,10^. However, clinically available methods for collagen analysis are rather limited being to a large extent restricted to the biopsy with further immunohistochemical analysis (IHC). The latter is expensive, time-consuming and requires complex multiple-step processing of tissue, staining, etc.^11^. Moreover, the preparation procedure may cause damage and/or modification of the tissue samples that may lead to misinterpretation of the of the obtained results^12,13^. Reproducibility, reliability, and accuracy of the results of histological analysis of collagen in tissue often suffers from the absence of objective quantitating in this approach.

Second Harmonic Generation (SHG) microscopy is a rapidly evolving approach for imaging tissues in situ. It is a powerful tool for the high-contrast, label-free, non-destructive study of biological objects with a sub-micron resolution^14^. SHG is a second-order non-linear optical process that enables visualizing non-centrosymmetric areas in the tissue samples^15,16^. It is also almost entiely free of phototoxicity and photobleaching^17^.

The second-order non-linear response of collagen^18,19^ originates from the peptide bonds of tightly aligned collagen triple helix^20^. This allows one to visualize collagen in the tissue via SHG without any specific labelling. High SHG signal intensity, contrast, and sub-micron resolution of images obtained by SHG microscopy provide a solid background for the effective analysis of collagen distribution/modification in lung tissue during PAH-associated fibrosis progression. Quantitative image processing allows minimizing the interpretation errors and makes it possible to study temporal evolution of fibrosis revealing the tissue changes that may be overlooked by conventional approaches. Collagen SHG image processing is often based on: (i) texture analysis (first and second order statistics F-/SOS)^9^, (ii) Fourier analysis^21^, and (iii) segmentation of individual fibers^22^, providing information on collagen content, its uniformity and density^23^, collagen fiber angle distribution^24^, shape^25^, network formation^26^, anisotropy of fiber orientation^27^, etc.

In this paper, we demonstrate the application of recently developed wide-field SHG microscopy^28^ for label-free investigation of fibrosis progression during PAH. In contrast to the laser-scanning SHG microscopy, its wide-field version allows obtaining tiled (2 × 2 mm^2^) high resolution SHG images of collagen distribution within a relatively short (less than 3 min) acquisition time. We reveal quantitative and qualitative changes in the collagen content, texture, and individual fiber structure in lung tissue slices of rats with monocrotaline (MCT)-induced PAH. We demonstrate that in rats with PAH, collagen content increases and fibers lengthen with time. Highly ordered fibers form a dense network both around blood vessels and in the surrounding tissue propagating deep inside the alveolar region. All the data obtained by SHG image processing using MatLab 2020 (MathWorks, USA) are fully supported by the results of the conventional IHC analysis of molecular markers of fibrosis progression (collagen I and III, tissue inhibitor of metalloptotease-1 (TIMP-1)). Although SHG is insensitive to the collagen type, SHG image visualizes the collagen fiber network transformations, which are of crucial importance for accurate diagnosis and prognosis of the disease development and can not be assessed by the IHC.

## Results and discussion

### IHC analysis of lung tissue during the PAH progression

Fibrillar collagen of type I and III comprise over 90% of the collagens in the lung parenchyma^29^. ECM remodeling during fibrosis is characterized by variations in its content^30^, and the increased collagen levels may indicate the stage of the pathology progression^31^. Metalloproteinases (MPP) are responsible for collagen degradation^32^, and the imbalance in MPP levels can contribute to fibrosis development during PAH^33^. TIMP-1 is an inhibitor of all types of MPP, responsible for the degradation of most fibrillar collagens. Thus, increased circulating level of TIMP-1 indicates the disruption of the collagen degradation and its accompanying accumulation.

In the present study, the 8-week model of PAH induced by MCT at a dose of 60 mg/kg was used, basing on the results obtained by our group in the previous studies of the dynamics of morphological changes in lung tissue and right ventricular myocardium^34^. It was revealed that 8 weeks after the administration of MCT, clinically characteristic pathohistological signatures of PAH were observed, including: (1) plexiform lesions, which most clearly illustrate vascular changes; (2) dilated lesions of the pulmonary arterioles; (3) classical arteritis with transmural inflammatory reaction and signs of fibrinoid necrosis^34^. Further studies of the processes of PAH-associated inflammation^35^, as well as the effect of atorvastatin on the process of remodeling of the right ventricle and pulmonary artery^36^ allowed substantiating the use of 8-week PAH model in the present study.

Although after MCT injection, the rat survival was 90%, 80%, 57%, and 20% in 2-, 4-, 6-, and 8-week groups, respectively, our previous data support the hypothesis that even on late stages of PAH progression the characteristic features vary and differ from those observed at the early stages of PAH. Some key results supporting the use of 8-week MCT-induced PAH model are presented in the Supplementary Material, Morphometric analysis and morpho-functional parameters, determined in animals with PAH.

**Figure 1:**
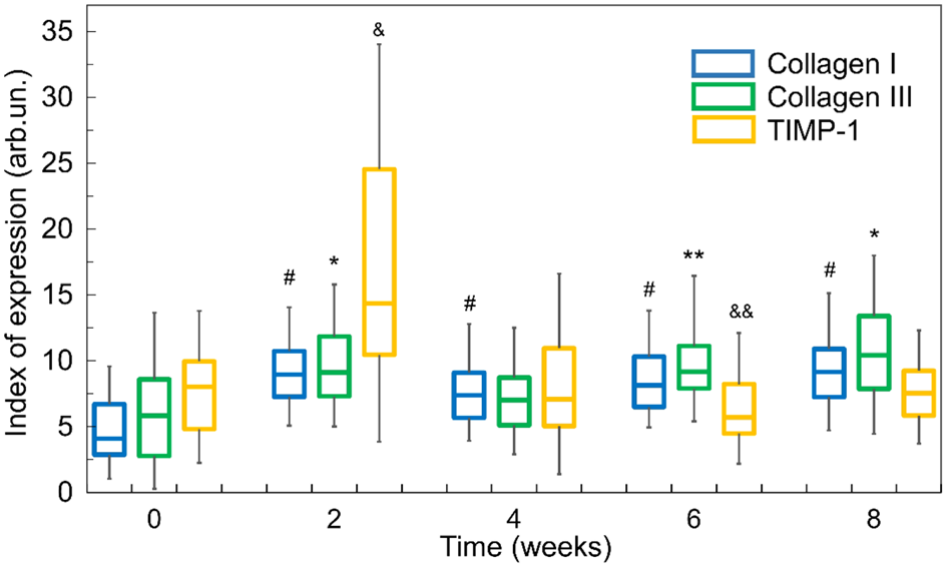
Expression indexes of collagen I, collagen III and TIMP-1 during PAH progression. Statistical difference: # *p* < 0.001 for collagen I; *p < 0.001 and **p < 0.01 for collagen III; ^&^*p* < 0.001 and ^&&^*p* < 0.01 for TIMP-1 as compared with corresponding control groups.

Quantitative analysis of IHC allows revealing the changes in the expression levels of collagen I and III, and TIMP-1, which are molecular markers of fibrosis development accompanying the PAH progression. Corresponding indexes of expression (IEs) are presented in Fig. 1; statistical significances for comparison of all experimental groups are summed up in Table S1 (Supplementary material).

As shown in Fig. 1, the expression of collagen I in the lung tissue of rats significantly increased in all experimental groups (2, 4, 6 and 8 weeks of PAH progression) compared with the control one.

The expression of collagen III is periodic. A significant increase in its quality is observed after 2 weeks of the PAH progression (p < 0.001). Then the normalization of collagen III levels to control values is registered with a further slight increase after 6 weeks of PAH progression (Fig. 1).

TIMP-1 expression is time-dependent with a twofold increase after 2 weeks of PAH progression in rats compared to the control group of healthy animals. However, in contrast to the increase in collagen I and III levels at the later stages of PAH, the TIMP-1 expression is downregulated to the control values throughout the remaining period of observation.

Figure 1 shows that fibrogenesis begins after 2 weeks of the MCT induced PAH. The maximum increase in TIMP-1 expression at the 2nd week of the experiment (p < 0.001 vs. all groups) promotes the synthesis activity of fibroblasts and a change in the balance between collagen I and III synthesis. Further progression of PAH (6–8 weeks) is characterized by a decrease in the expression of the MPP inhibitor (p < 0.001), an increase in the type III collagen synthesis from 7.0 to 10.41 (p < 0.001) from 4 to 8th weeks of the PAH. As a result of ECM remodeling, in the local microenvironment of the pulmonary arteries, a permissive environment is formed for the initiation of fibrogenesis in the vascular wall.

### SHG imaging of rat lung tissue during PAH progression

**Figure 2:**
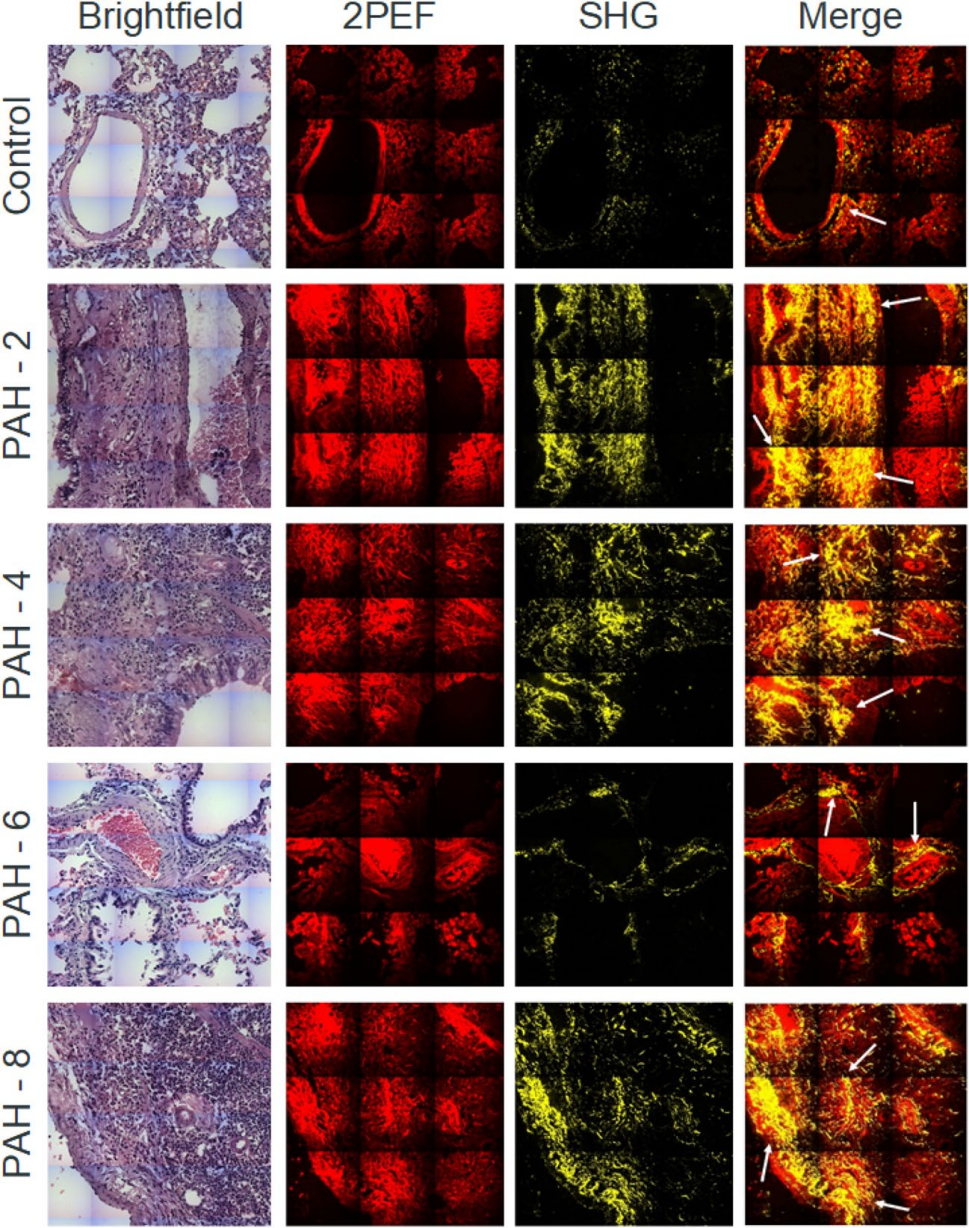
Brightfield images of HE-stained lung tissue, 2PEF, SHG and 2PEF/SHG merged images. Data are presented for lung tissue of control group, and of rats on the 2nd, 4th, 6th, and 8th week of PAH progression. Image size is 450 × 450 µm^2^.

Combination of SHG and two-photon excitation fluorescence (2PEF) imaging allows visualization of both the fibrotic structures and external tissue and, thus, provides a complete picture of the collagen fiber branching and enlargement inside the tissue^37^. Endogenous 2PEF signal often originates from such metabolic compounds as of the nicotinamide adenine dinucleotide, the flavins, the lipopigments, the porphyrins, etc.^38^ In our case, hematoxylin–eosin (HE)-staining, applied for simultaneous brightfield microscopy, also contributes to 2PEF signal. Images of HE-stained slices of lung tissue are presented in Fig. 2 with SHG of collagen colored yellow and 2PEF—red. In the control sample, collagen can be seen to surround the blood vessel wall (marked with arrows in Fig. 2) but is absent in the lung tissue. The images of HE-stained PAH demonstrate only thickening of blood vessel walls compared to the control sample, while the SHG/2PEF images demonstrate significant collagen overproduction. One can observe from Fig. 2 that collagen content increases with time and that highly assembled long fibers form dense networks both around the blood vessels and in the surrounding tissue, thus, propagating deep inside the alveolar region (marked with arrows, Fig. 2). This supports our previous results^35^ and IHC data, indicating a significant increase in the expression of collagen after 4–8 weeks of PAH progression.

### Anisotropy of collagen fiber orientation during PAH-associated fibrosis

**Figure 3:**
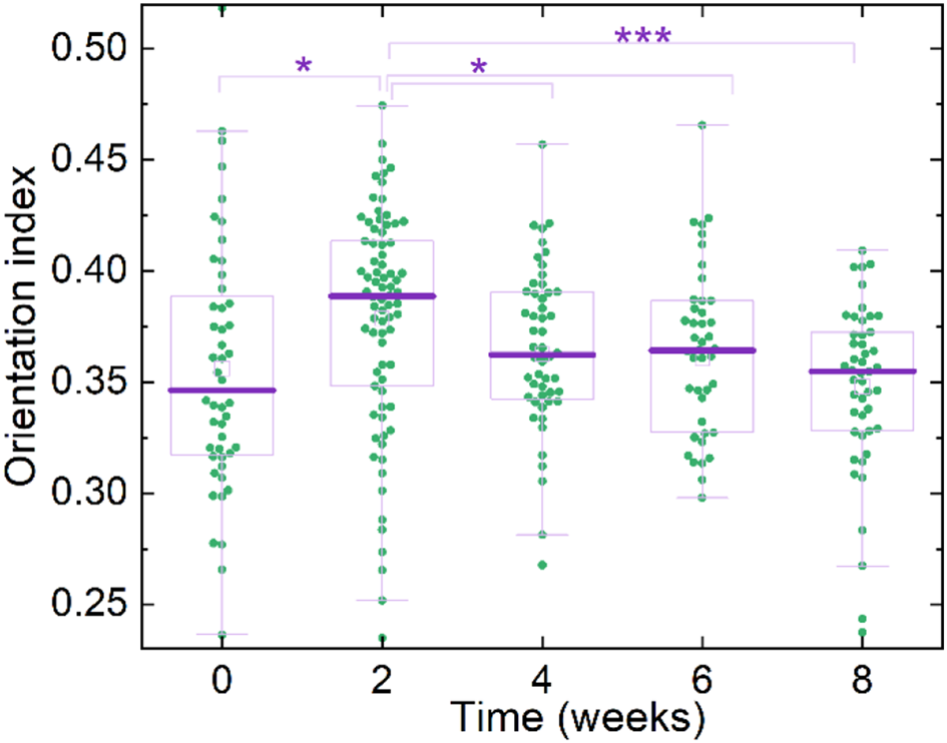
OI values calculated on SHG images of control samples (0 weeks) and during the PAH progression. The statistical significance of the difference of different distributions is *p* < 0.1*, and *p* < 0.001***.

Information on anisotropy of collagen fiber orientation is extracted from SHG images by fast Fourier transform (FFT) analysis. The temporal evolution of orientation index (OI) calculated using Eq. (2) is presented in Fig. 3. The lower the OI, the poorer the ordering of collagen fibers. Isotropic collagen structure is characterized by OI = 0 and a circular FFT image^39^. On the other hand, the higher the OI, the more pronounced is the anisotropy of collagen fiber orientation. The lung tissue of healthy rats was found to have lower OI than that during PAH progression. This indicates that PAH is associated with ordering of collagen fibers. By the second week of PAH-associated development of fibrosis, OI increases significantly, which likely indicates fiber elongation and their protrusion in the lung tissue. Subsequent decrease of OI indicates collagen fiber reorganization and the formation of more isotropic collagen network.

### Texture analysis of SHG images

#### First order statistics

The different FOS parameters yield collagen content^9^, its uniformity, and density^23^. Specifically, the mean and standard deviation relate to the amount of collagen and homogeneity of its distribution in the sample. Skewness is a measure of asymmetry of the distribution of pixel intensities. Kurtosis indicates how closely the distribution of pixel intensities resembles the normal distribution. Strong SHG signals usually result in a wide distribution of pixel intensities, and consequently, low value of kurtosis^9^.

**Figure 4:**
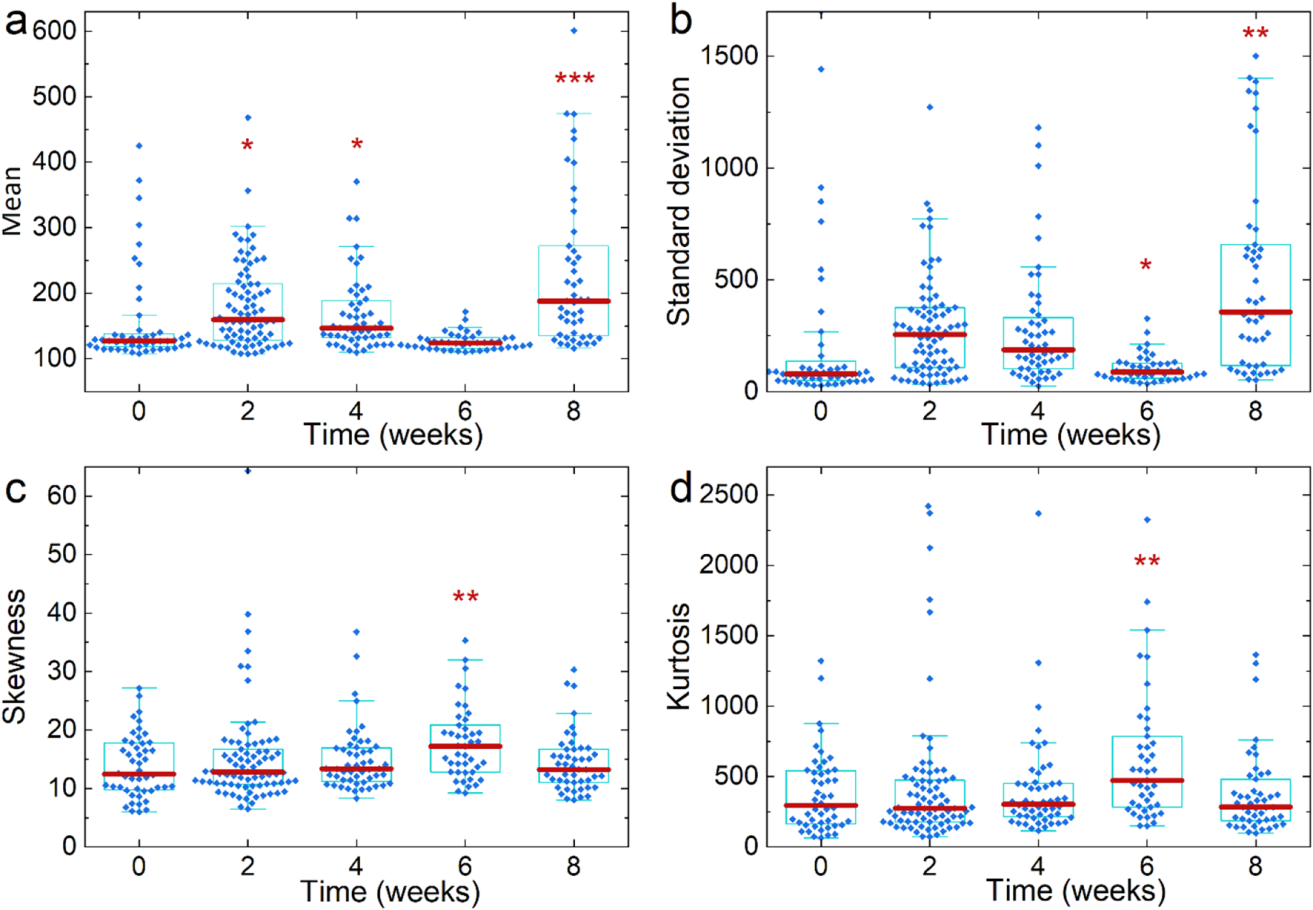
FOS parameters determined inform SHG images of control samples and during the PAH progression. (**a**) Mean, (**b**) standard Deviation, (**c**) skewness, (**d**) kurtosis. The statistical significance of the difference of different distributions relative to the control is *p* < 0.1*, *p* < 0.01**, *p* < 0.001***.

Distributions of mean and standard deviation are presented in Fig. 4a,b. The increase of both parameters at the early stage of the disease progression (2 weeks) indicates an increase of collagen production and growing heterogeneity of collagen distribution in the tissue. Both parameters decrease on the 4th and 6th week, and then sharply rise at the terminating stage of the PAH progression by the 8th week. The reduction of the collagen content after the 2nd week may indicate the organism struggling to dampen the effect of rapid PAH progression and restore the balance of collagen synthesis/degradation. This hypothesis is supported by the data of IHC and fully reiterates the evolution of the amounts of collagen I, collagen III, and TIMP-1.

The kurtosis and skewness values remained constant throughout the experiment and coincided with the values in the control group, with the exception of the 6th week of PAH progression, when an increase in these parameters was observed (see Fig. 4c,d).

#### Second order statistics

**Figure 5:**
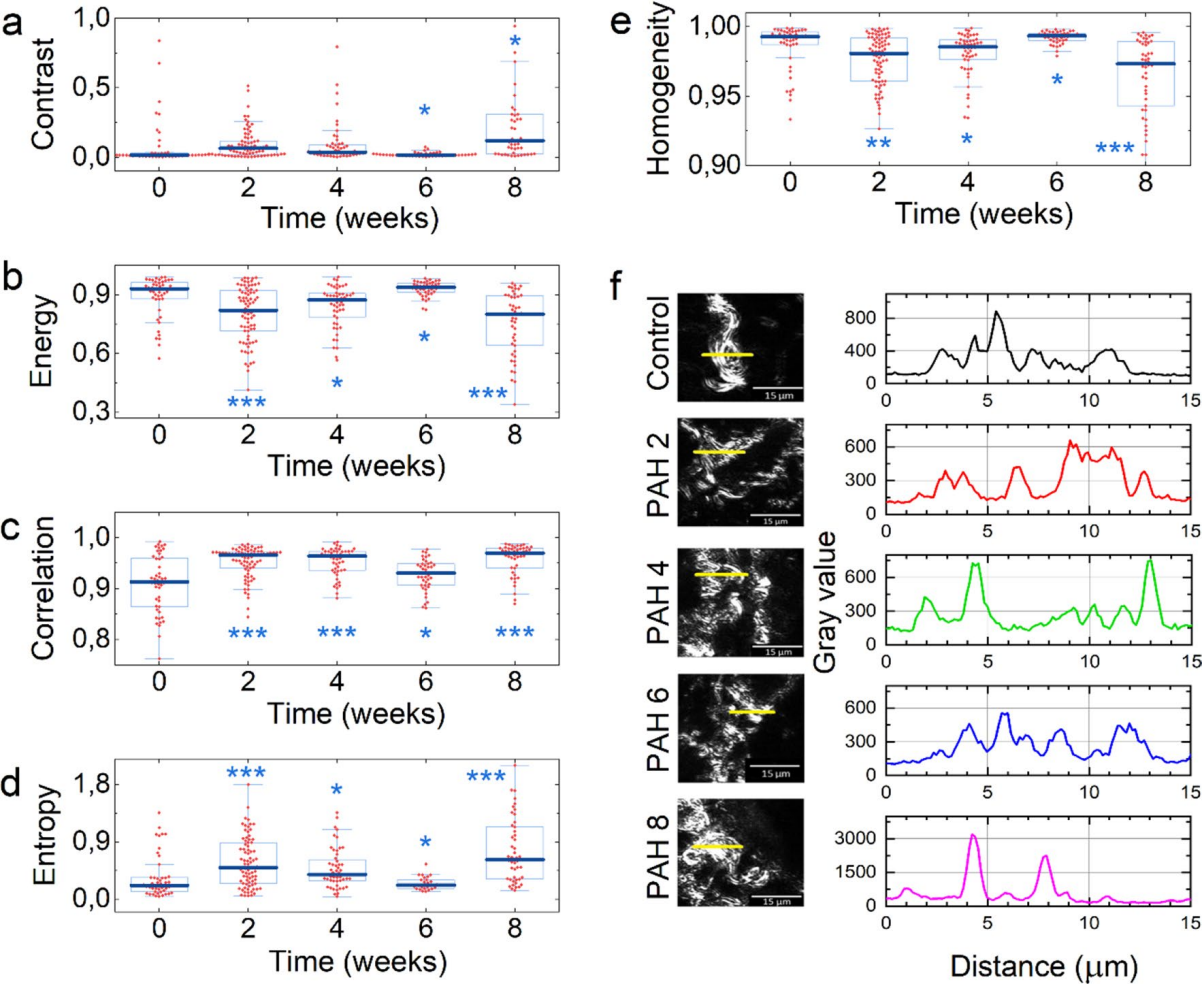
SOS parameters calculated on images of control samples of healthy lung tissue and at different stages of PAH progression. (**a**) Inertia (contrast), (**b**) energy, (**c**) correlation, (**d**) homogeneity, (**e**) entropy, (**f**) typical SHG collagen structures (scale bar 15 μm). The statistical significance of the difference of distributions relative to the control is *p* < 0.1*, *p* < 0.01**, *p* < 0.001***). Regions of interest (ROIs) size is 150 × 150 μm^2^.

SOS parameters are extracted from the gray level co‑occurrence matrix (GLCM) and provide information on the collagen distribution, spatial fiber organization, uniformity, etc. All calculated SOS parameters are presented in Fig. 5.

Inertia (or contrast), calculated for control and PAH samples at different stages, are presented in Fig. 5a. There is an increase in the value of inertia at an early stage of PAH progression (the 2nd week), indicating the rise of the number of the areas of high contrast compared to the control. This is likely associated with the formation of highly dense collagen bundles and the appearance of foci of collagen synthesis. A subsequent drop of contrast indicates homogenization of collagen distribution during the 4th and 6th week of PAH progression. However, the terminating phase of PAH is accompanied by a significant increase of the number of high contrast areas and occurs due to thickening of collagen bundles.

Calculated energy is presented in Fig. 5b. Energy values may vary from 0 to 1. The absence of dominant gray levels in the image corresponds to low energy values. Thus, low energy at the 2nd and 8th week depicts collagen fiber ordering and spreading.

Correlation is a measure of the presence of periodic structures in an image. Zero correlation arises from a uniform image^9,40^. Calculated correlation of SHG images is presented in Fig. 5c. Here, correlation is within the range of 0.9–1.0, indicating the presence of some periodic structures, both, it the case of healthy rats in the control group and rats with PAH at different stages of progression. Examples of such periodic structures are presented in Fig. 5f. These data indicate that despite the changes of the anisotropy of fiber orientation (Fig. 3), collagen packing into fibers characterized by periodicity is not significantly disrupted during the PAH progression according to small variations in correlation values. Nevertheless, the latter are significantly distinguishable (Fig. 5c).

The evolution of entropy values (Fig. 5d) correlates with the data discussed above. Low entropy of control samples and lung tissue at the 6th week of PAH-associated fibrosis development indicates the less clearly separated fibers in the selected area in comparison with other stages of desease progression. This could be caused, e.g., by the fiber swelling during inflammation on the 6th week of PAH progression^41^. High entropy values on the 2nd, 4th, and 8th weeks of PAH are caused by bright and distinct but not necessarily ordered collagen fibers standing out from a homogeneous background.

IDM is a measure of how tightly GLCM elements are distributed to its diagonal. IDM equals 1 for a diagonal GLCM matrix. Thus, IDM measures the amount of local uniformity present in the image and is a metric of pixel similarity to its neighbours^9^. The higher is the IDM value, the denser or thicker are the collagen fibers. A decrease in the IDM values during the PAH progression (Fig. 5e) indicates the spreading of the network of thin and disordered collagen fibers and the development of fibrosis. These data are in good agreement with Energy values, confirming the growth of collagen fibers into the lung tissue both near and away from the walls of blood vessels.

Physiologically, the observed collagen changes during 5-week period of PAH progression among other things are associated with gradual accumulation in lung tissue of inflammatory markers including monocyte chemoattractant protein 1 (MCP-1) and interleukin-6 (IL-6) as demonstrated in^42^. Treatment with silibinin (C-X-C chemokine receptor type 4 inhibitor) reduces the PAH symptoms during the first two weeks due to the downregulation of the gene expression of inflammatory markers in pulmonary arteries but not in the lung tissue. However, silibinin fails to ameliorate PAH at later stages. At that time, it becomes an irreversible condition because of MCP-1 and IL-6 accumulation in the tissue. Increased MCP-1 induces collagen synthesis by lung fibroblasts and also recruits monocytes to the sites of inflammation^43^. Heightened IL-6 induces collagen I production by dermal fibroblasts^44^ and contributes to ECM deposition^45^.

On the basis of our results of SHG image analysis, taking into account the physiological underpinning, the following stages of PAH development/progression may be proposed:

(i) the pathology develops rapidly in the beginning: by the 2nd week, the mean, standard deviation, OI, inertia and entropy increase, but energy and homogeneity decrease, which indicates collagen accumulation, collagen fiber elongation and their protrusion in the lung tissue;
(ii) the organism tries to regulate the progression of the disease: by the 4th week, the drop of mean and standard deviation, high entropy values designate the possible inflammation-mediated disruption of collagen structure as well as the activation of the collagen degradation system intended to correct the imbalance in collagen synthesis/degradation;
(iii) phantom recovering of the organism by the 6th week, as the mean and standard deviation drop, entropy is low; however, an increase of kurtosis and skewness evidence significant collagen redistribution that indicates collagen fiber thickening and penetration deep into the lung tissue and thus might be a point-of-no-return in PAH pathogenesis;
(iv) finally, by the 8th week of pathology, a total tissue failure arises which is the outcome of a significant increase in collagen content and thickening of collagen bundles characterised by the increase of the mean, standard deviation, inertia, entropy and the decrease of the energy and homogeneity.

Overall, SHG image processing provides a complete picture of morphological collagen modifications during the PAH-associated progression of fibrosis. The evolution of different FOS and SOS parameters point to the same characteristic changes of collagen fiber structure and network organization that also agree with the results of IHC.

To sum up, our home-made microscope system provides some prominent advantages. First, it is a two-in-one system, which combines 2PEF and SHG microscopes. This allows simultaneous visualization of collagen structures and surrounding tissue as an endogenous 2PEF signal originates from such metabolic compounds as the nicotinamide adenine dinucleotides, the flavins, lipopigments, the porphyrins, etc.^46^ which are components of the lung tissue. Second, this system allows one to obtain large tiled high resolution SHG images during less than 3 min without applying any labels and additional staining of the tissues. It minimizes time to create the large datasets for further post-processing. Used approach combines various methods of qualitative and quantitative data processing, which allows us to conduct an effective analysis of collagen distribution/modification in lung tissue during fibrosis pathogenesis. Moreover, the applying of mathematical algorithms to obtained SHG image datasets for their quantitative processing eliminates the errors associated with the human factor and permits extracting more valuable information.

It is worth noting that our approach has several limitations. They include: (i) inability to recognize different types of collagen, as it requires employing SHG polarization anisotropy and forward – backward SHG detection^47^, while our approach is based on the wide-field forward SHG detection; (ii) the need to take a biopsy or obtain tissue material surgically, which makes the method invasive; (iii) to date the collected database needs to be expanded, as it is necessary for training the neural network for fully automated recognition of the stages of fibrosis progression. This is, however, beyond the scope of the present work. With the accumulation of more data on a larger set of samples, SHG image analysis is expected to provide reliable signatures for the various stages of PAH pathogenesis.

This is beyond the scope of the present work, but with the accumulation of more data on a larger set of samples, SHG image analysis may prove to also provide reliable signatures for the various stages of PAH pathogenesis.

## Conclusion

In the present work, we employed the wide-field SHG microscopy to visualize the evolution of collagen structures in the lung tissue of rats with induced PAH. By analysing statistics and Fourier transforms of the SHG image textures we were able to reveal characteristic changes of collagen morphology and content, and interpreted them in terms of different stages of PAH. Our results pave the way towards SHG imaging-based diagnosis of PAH that can be implemented with the machine learning using the data sets accumulated for the algorithm training. Since the SHG imaging is relatively fast, label-free and non-destructive, such a neural network based analysis has a potential of becoming a technique of choice for the investigation of PAH-associated progression of fibrosis free from the error-prone human interference. Moreover, this method possesses high potential to be used in a wider range of lung diseases or conditions, e.g., for assessing pulmonary fibrosis in interstitial lung disease, after lung(s) transplantation, cancer, etc., if a biopsy is taken or the surgical material is available, because clinically relevant methods for assessing fibrosis are currently limited.

## Methods and materials

### PAH model in rats

The studies were carried out on 64 outbred white rats (males, 200–250 g) purchased from the Experiment Animal Center of Belarusian Medical Academy of Postgraduate Education. PAH in rats was chemically induced via MCT (Sigma-Aldrich, USA) injections at a dose of 60 mg/kg according to the procedure described in^48^. All animal experiments were performed in accordance with the principles of bioethics. In the course of the study, we were guided by compliance with the requirements of the European Convention for the protection of vertebrate animals used for experiments or other scientific purposes, as well as the requirements and recommendations, regulatory, scientific, methodological and reference materials for keeping, feeding and removing them from the experiment with subsequent disposal. This study was approved by the Ethics Commission of the Belarusian Academy of Postgraduate Education (Approval number 4 from 23.09.2020) and was carried out in accordance with the recommendations in the ARRIVE guidelines^49^.

After MCT injection, the rats of the PAH group were randomly divided into 4 groups: 2 week, 4 week, 6 week, and 8 week groups. The results were compared with corresponding data obtained for healthy animals.

### Lung tissue preparation

Slices of the lung tissue were fixed in 10% neutral formalin for 48 h. Then they were washed in water flow for 24 h, dehydrated in ethanol of increasing concentrations (70%, 80%, 90%, absolute ethanol). Then the tissue samples passed through ethanol-xylene, xylene, xylene-paraffin and, finally, were embedded in paraffin. Slices of 3–4 μm thickness of the prepared tissue were stained with HE^50^.

### Immunohistochemical study

The study of the tissue slices was performed by the analysis of micrographs obtained using light microscopes Motic (Motic, China) and DMLS (Leica, Germany).

Immunohistochemical study of the expression levels of molecular markers (collagen I, III and TIMP-1) was carried out using the following monoclonal antibodies: (i) polyclonal rabbit IgG for Collagen type I (Abcam, Great Britain), (ii) polyclonal rabbit IgG for Collagen type III (Thermo Fisher scientific, USA), (iii) monoclonal mouse IgG1 for TIMP-1 (Thermo Fisher scientific, USA).

All IHC- staining was performed according to commonly used protocols described in^51,52^.

For IHC studies, tissue samples were deparaffinized in xylol by two-step washing for 10–15 min for each step. Then the slices were rehydrated in alcohols of decreasing concentrations followed by washing in distilled water. Then heat-induced epitope retrieval was performed in a microwave oven (Samsung, China) in 0.01 M citrate buffer (Carl Roth GmbH + Co. KG, Germany, pH 6.0) for 10 min, preheating the retrieval buffer in accordance with the standard protocol as described in^53^. After that, the IHC staining was performed according to the following protocol. Incubation with primary antibodies was carried out in a humid chamber for about 1–2 h at 37 °C or 24 h at 4 °C. Then, the slices were treated by Polymer Helper and Polyperoxidase-Anti-Mouse/Rabbit IgG, containing a complex of secondary antibodies and chromogen diaminobenzidine (DAB, Elabscience, China), at 37 °C for 20 and 30 min, respectively. After each step, the slices were rinsed in phosphate-buffered saline. After staining with DAB, sections were counterstained with HE.

Then the slices were placed in absolute ethanol two times for 7 min, afterwards in xylene two times for 10 min, and then the slices were embedded in Canadian balsam (AppliChem, Spain). To control the activity of primary antibodies (to exclude false positive and false negative results), one negative and one positive control staining were performed in each experimental series. As a negative control, the slices pretreated with 1% bovine serum albumin solution (Helix, Russia) instead of incubation with the primary antibody were used. For positive control, lung (TIMP-1), kidney (collagen I), and ovarian (collagen III) tissues were examined.

### Conventional quantitative IHC micrograph analysis

The quantitative assessment of the expression of biomolecular markers was carried out by analyzing digital images obtained with Leica DMLS microscope using the pre-installed software and a JVC digital camera (at 400 × magnification, at least 30 view areas). The positive pixel count algorithm and software for morphometry AperioImageScope12.1.0.5 were applied.

The image analysis yielded data on the prevalence and intensity of the brown color of the DAB reaction products in tissue slices. Digital images of non-overlapping areas with clearly defined nuclei, cells and vessels of the lungs were selected. The IEs of biomolecular markers was calculated using the formula:

1 $$IE=\frac{\text{Number of positive pixels}}{\text{Total number of pixels}}\times 100 .$$

### SHG and fluorescence microscopy

Lung tissue samples of healthy animals (control group) and animals with PAH on the 2nd, 4th, 6th and 8th week of disease progression were investigated by a combination of brightfield, 2PEF and SHG microscopies of HE-stained samples.

All imaging was performed on a custom-built wide-field non-linear microscopy setup based on a modular microscope (Applied Scientific Instruments, USA), as described previously^28^. In brief, a sample area of about 150 × 150 µm^2^ was illuminated with FemtoLux3 laser (Ekspla, Lithuania) with 1030 nm wavelength, 262 fs pulse duration, 1 MHz pulse repetition frequency, and 1.5 W of average power at the sample. The resulting signal was detected in the forward direction with Neo 5.5 sCMOS (Andor Technology Ltd, UK) with 40 × magnification. SHG and fluorescence signals were separated by band-pass optical filter FF01-513/13–25 (Semrock, USA) and long-pass optical filter ET525lp (Chroma Technology GmbH, Germany), respectively. Tiled images of 150 × 150 µm^2^ sample areas were obtained by scanning the sample with a motorized mechanical stage. Both, SHG and fluorescence image integration time was 0.5 s. It took about 2.6 min to scan a tiled image. Colored brightfield transmission images were recorded on the same setup with DCC1645C-HQ CMOS camera (Thorlabs Inc., USA).

### SHG image analysis

**Figure 6:**
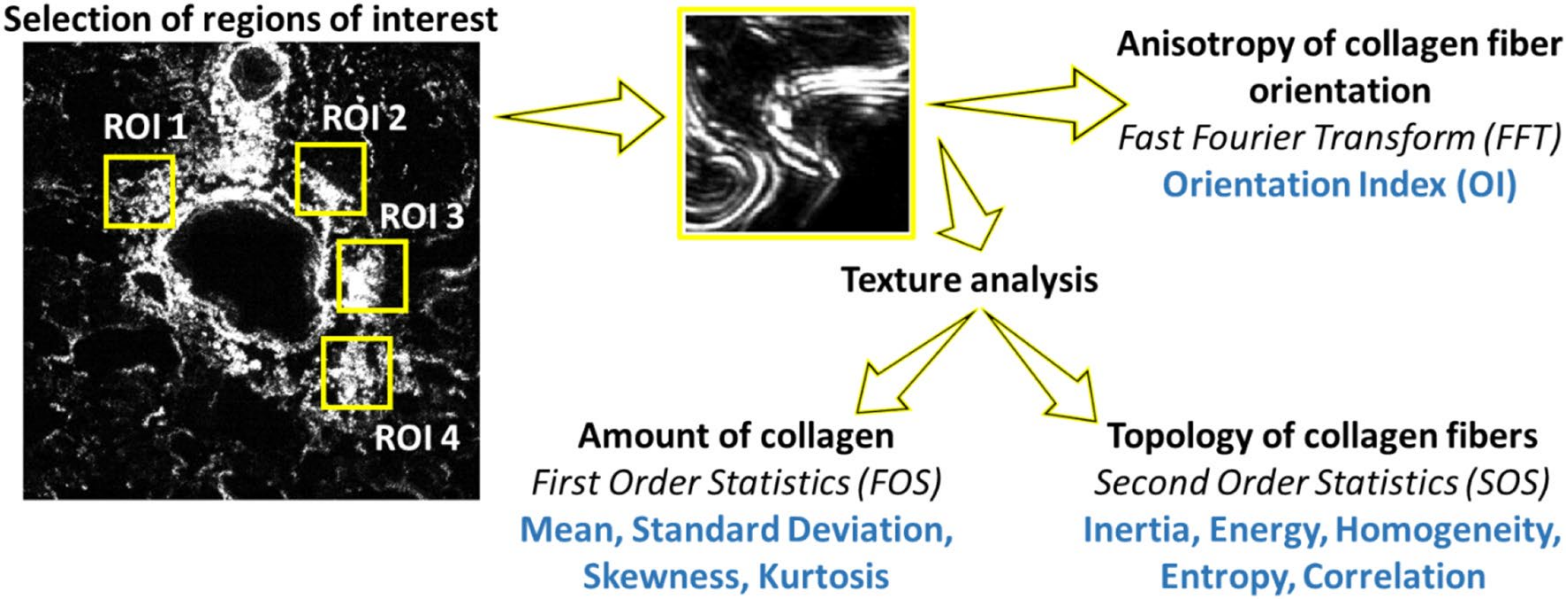
Procedures of SHG image analysis.

SHG images were examined over the manually selected ROIs. ROI images were quantitatively characterized using the FFT and texture analysis. Texture analysis consisted of FOS and SOS of GLCM (Fig. 6).

#### ROI selection rule

Square 150 × 150 μm^2^ ROIs were selected to be not further than 500 μm from the blood vessel wall (see Fig. S1, Supplementary material). For each experimental group (samples taken from healthy animals and animals at different stages of PAH progression) 50–80 non-overlapping ROIs were selected (specific numbers of ROIs for each experimental group are given in Table S3, Supplementary material).

#### FFT analysis

The anisotropy of the collagen fiber network was quntified by the OI of all ROI images. The OI was obtained by shot and long axis of the binarized with threshold of 0.38 FFT of a ROI image (see Supplementary material)^21^:

2 $$OI = \left[1-\left(\frac{\text{short axis}}{\text{long axis}}\right)\right].$$

That is the *OI* = 0 and *OI* = 1 correspond to the randomly and perfectly oriented collagen fibers, respectively.

#### Texture analysis (FOS/SOS)

FOS relates to the distribution of pixel intensity values irrespective of their position in an image. Its parameters are mean, standard deviation, skewness (asymmetry), and kurtosis of the intensity distribution histogram:

Mean:

3 $$\mu = \frac{1}{{n}^{2}}\sum_{i,j}{I}_{ij},$$

Standard deviation:

4 $${\sigma }^{2}=\frac{1}{{n}^{2}}\sum_{i,j}{\left({I}_{ij}-\mu \right)}^{2} .$$

Skewness:

5 $$s=\frac{\frac{1}{{n}^{2}}\sum_{i,j}{\left({I}_{ij}-\mu \right)}^{3}}{{\left(\sqrt{\frac{1}{{n}^{2}}\sum_{i,j}{\left({I}_{ij}-\mu \right)}^{2}}\right)}^{3}} .$$

Kurtosis:

6 $$k=\frac{\frac{1}{{n}^{2}}\sum_{i,j}{\left({I}_{ij}-\mu \right)}^{4}}{{\left(\frac{1}{{n}^{2}}\sum_{i,j}{\left({I}_{ij}-\mu \right)}^{2}\right)}^{2}} .$$

Here, *n* is the image size in pixels, and ${I}_{ij}$ is the pixel intensity at $(i,j)$.

SOS relates topologies from different image areas. SOS parameters are obtained from GLCM ${P}_{ij}$^54^, that represents the probability of occurrence of pixel pairs with specific values and specified spatial relationship (for the calculation of ${P}_{ij}$ see formula (S1) in Supplementary material).

SOS parameters are the following^54^:

Energy (angular second moment, Uniformity) is a measure of uniformity of the distribution of gray levels in an image:

7 $$\mathrm{Energy }= \sum_{i,j}{{P}_{ij}}^{2} .$$

Inertia (Contrast) is a measure of pixel similarity to its neighbours l and is sensitive to differences within the GLSM:

8 $$\mathrm{Inertia }= \sum_{i,j}{\left(i-j\right)}^{2}{P}_{ij}.$$

Correlation is a measure of a linear dependence of gray levels of neighboring pixels:

9 $$\mathrm{Correlation }= \sum_{\mathrm{i},\mathrm{j}}\frac{\left(\mathrm{i}-{\upmu }_{\mathrm{i}}\right)(\mathrm{j}-{\upmu }_{\mathrm{j}}){P}_{ij}}{{\upsigma }_{\mathrm{i}}{\upsigma }_{\mathrm{j}}} .$$

The inverse difference moment (IDM), or Homogeneity, is a measure of local homogeneity of an image:

10 $$\mathrm{IDM}= \sum_{\mathrm{i},\mathrm{j}}\frac{{P}_{ij}}{1+{\left(\mathrm{i}-\mathrm{j}\right)}^{2}}$$

Entropy is a measure of image uniformity:

11 $$\mathrm{Entropy}= -\sum_{i,j}{P}_{ij}\cdot \mathrm{Ln}\left({P}_{ij}\right)$$

### Statistics

The statistical significance of the difference between the distributions of various parameters calculated on images from healthy control group and PAH animals was performed by statistical analysis of variance (one-way ANOVA) by applying an unpaired two-tailed Student’s T-test in Microsoft Office 365 Excel (Microsoft Corporation, Redmond, Washington, DC, USA). The significance of differences between all statistical parameters of ROIs of experimental groups of animals with PAH and control group of healthy animals is presented in Table S2 (see Supplementary material).

Distributions of parameters obtained from IHC, FFT, SOS and FOS are represented by Beeswarm box plot. The 75th and 25th percentiles are denoted as the top and bottom of each rectangular box, respectively. The median is shown inside the box. The whiskers are plotted as 1.5 times of interquartile range below and above the box.

## Supplementary Information

Supplementary Information.

## Data Availability

The data that support the findings of this study are available from the corresponding author upon reasonable request.
