# Supplementary material for: Quantitative and qualitative analysis of pulmonary arterial hypertension fibrosis using wide-field second harmonic generation microscopy
Source: Sci Rep. 2022 May 5;12:7330. doi: 10.1038/s41598-022-11473-5 (PMC9072392; doi:10.1038/s41598-022-11473-5)
Supplement: Supplementary file 1 — Supplementary Information. [file 41598_2022_11473_MOESM1_ESM.pdf]

Scientific Reports

Supplementary material

Quantitative and qualitative analysis of pulmonary arterial hypertension fibrosis using wide-field second harmonic generation microscopy

Yaraslau Padrez[1,2,*], Lena Golubewa[1,2], Tatsiana Kulahava[2], Tatyana Vladimirskaja[3], Galina Semenkova[4], Igor Adzerikho[5], Olga Yatsevich[5], Nadezda Amaegberi[4], Renata Karpicz[1], Yuri Svirko[6], Polina Kuzhir[6], Danielis Rutkauskas[1]

[1]Center for physical sciences and technology, Vilnius, Lithuania
[2]Institute for nuclear problems of Belarusian state university, Minsk, Belarus
[3]Central Scientific and Research Laboratory of BelMAPE, Minsk region, Belarus
[4]Belarusian State University, Minsk, Belarus
[5]State Educational Establishment BelMAPE, Minsk, Belarus
[6]University of Eastern Finland, Department of Physics and Mathematics, Institute of photonics, Joensuu, Finland

Table S1. Indexes of expression (IE) of molecular markers during PAH progression

Groups of experimental animals		IE, % (25;75)	95% Confidence Interval	P*
**Collagen I**				
1	Control	4.07 (2.86; 6.70)	1.83-2.87	$P_{1-2} < 0.001$ $P_{1-3} < 0.001$ $P_{1-4} < 0.001$ $P_{1-5} < 0.001$
2	2 weeks of PAH	8.96 (7.27; 10.73)	2.21-3.33	$P_{2-3} = 0.58$ $P_{2-4} = 0.51$ $P_{2-5} = 0.89$
3	4 weeks of PAH	7.39 (5.67; 9.09)	1.76-3.70	$P_{3-4} = 0.21$ $P_{3-5} = 0.10$
4	6 weeks of PAH	8.13 (6.49; 10.31)	1.57-3.49	$P_{4-5} = 0.51$
5	8 weeks of PAH	9.14 (7.25; 10.90)	2.54-4.22	
**Collagen III**				
1	Control	5.83 (2.77; 8.58)	2.48-5.07	$P_{1-2} < 0.001$ $P_{1-3} = 0.78$ $P_{1-4} = 0.03$ $P_{1-5} < 0.001$
2	2 weeks of PAH	9.11 (7.29; 11.83)	2.27-3.95	$P_{2-3} = 0.001$ $P_{2-4} = 0.61$ $P_{2-5} = 0.14$
3	4 weeks of PAH	7.00 (5.11; 8.72)	2.22-3.78	$P_{3-4} < 0.01$ $P_{3-5} < 0.001$
4	6 weeks of PAH	9.17 (7.89; 11.11)	2.48-5.35	$P_{4-5} = 0.47$
5	8 weeks of PAH	10.41 (7.87; 13.39)	3.42-4.61	
**TIMP-1**				

1	Control	8.01<br>(4.81;9.95)	2.57-3.83	$P_{1-2} < 0.001$<br>$P_{1-3} = 0.89$<br>$P_{1-4} = 0.03$<br>$P_{1-5} = 0.69$
2	2 weeks of PAH	14.35<br>(10.5; 24.53)	6.60-9.49	$P_{2-3} < 0.001$<br>$P_{2-4} < 0.001$<br>$P_{2-5} < 0.001$
3	4 weeks of PAH	7.07<br>(5.04; 10.95)	3.65-5.65	$P_{3-4} = 0.068$<br>$P_{3-5} = 0.97$
4	6 weeks of PAH	5.70<br>(4.48; 8.22)	2.30-3.89	$P_{4-5} < 0.001$
5	8 weeks of PAH	7.53<br>(5.84; 9.23)	2.13-3.07	

Notes:

*Statistical difference between the experimental groups was calculated by one-way ANOVA applying an unpaired two-tailed Student's T-test

Table S2. The significance difference between statistical parameters of ROIs of experimental groups of animals with PAH and control group of healthy animals, performed by one-way ANOVA applying an unpaired two-tailed Student's T-test

	**Mean**	**Standard deviation**	**Skewness**	**Kurtosis**	**Contrast**	**Correlation**	**Energy**	**Homogeneity**	**Entropy**	**OI (FFT)**
**Con. - PAH 2**	<0,1	0,551	0,285	0,308	0,717	<0,001	<0,001	<0,01	<0,001	<0,1
**Con. - PAH 4**	0,366	0,669	0,302	0,691	0,615	<0,001	<0,1	<0,1	<0,1	0,510
**Con. - PAH 6**	<0,01	<0,1	<0,01	<0,01	<0,1	<0,1	<0,1	<0,1	<0,1	0,687
**Con. - PAH 8**	<0,001	<0,01	0,566	0,926	<0,1	<0,001	<0,001	<0,001	<0,001	0,418
**PAH 2 - PAH 4**	0,245	0,912	0,792	0,382	0,704	0,869	<0,1	0,130	0,127	<0,1
**PAH 2 - PAH 6**	<0,001	<0,001	<0,1	0,627	<0,001	<0,001	<0,001	<0,001	<0,001	<0,1
**PAH 2 - PAH 8**	<0,01	<0,001	0,532	0,297	<0,001	0,260	<0,1	<0,1	<0,1	<0,001
**PAH 4 - PAH 6**	<0,001	<0,001	<0,1	<0,1	<0,01	<0,001	<0,001	<0,001	<0,001	0,810
**PAH 4 - PAH 8**	<0,01	<0,01	0,634	0,637	<0,01	0,259	<0,01	<0,001	<0,01	<0,1
**PAH 6 - PAH 8**	<0,001	<0,001	<0,01	<0,01	<0,001	<0,001	<0,001	<0,001	<0,001	0,162

ROI selection

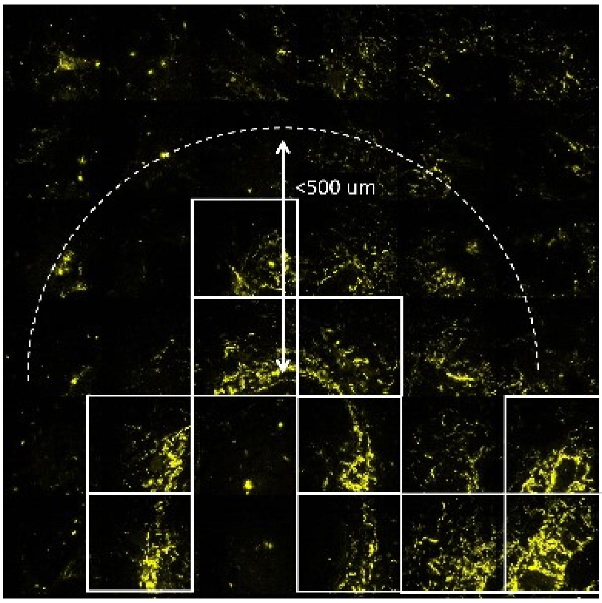

Figure S1. Scheme of ROI selection in large-scale SHG images. Dashed line indicates the annular region for ROI selection around the vessel, squares indicate the ROIs.

Table S3. Number of ROIs selected for analysis

Selection of ROI	
**Experimental group**	**Number of ROIs**
Control	48
PAH 2 weeks	78
PAH 4 weeks	52
PAH 6 weeks	43
PAH 8 weeks	47
**All**	**268**

FFT method description

OI is calculated through the long-to-short ellipse axis ratio obtained via FFT analysis of the ROI [S1]. The transformed image from each ROI was binarized applying threshold, computed by the Otsu method [S2]. Despite the presence of some residual noise, a binary image with a significant elliptical structure was obtained. A predefined 2-D circular averaging filter for remaining noise removal (radius = 3 px) was applied. The received ellipse was approximated by second order curve using a least squares fitting method [S3].

1. Obtaining Fourier Transform of an image.
2. Obtaining the centered spectrum (Fig. S2a-b).
3. Binarization by thresholding level [S2] (Fig. S2c).
4. 2-D circular averaging filter (Fig. S2d).
5. Determine the coordinates of the edge points of the ellipse.
6. Transfer of the origin of the reference system to the center of the ellipse.
7. Finding the ellipse equation by the Fitzgibbon method [S3] (Fig. S2e).
8. Finding the invariants of the curve of the second order.
9. Using the relations between the invariants and the semi-axes of the ellipse, Calculate "OI = 1-Semi-minor Axis / Semi-major Axis" (Solid red lines on Fig. S2e).

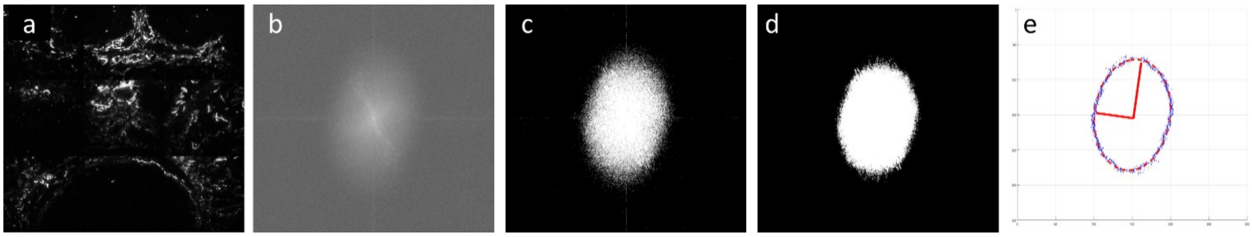

Figure S2. FFT processing and ellipse parameters calculation: a – image of selected ROI, b – FFT of the image, c – result of binarization, d – 2-D circular averaging filter implementation, e –second order curve approximation.

Gray-Level Co-occurrence Matrix for SOS parameters evaluation

In this study, the image intensity is quantified by 256 levels. The 256×256 Gray-level Co-occurrence Matrix (GLCM) $P(i,j,d)$ shows how often two pixels of *i-th* and *j- th* levels occur in the image at distance *d*. For a rectangular image having $N_x \times N_y$ pixels the GLCM is defined by the following equation [S4]:

$$P(i,j,d) = \sum_{x=1}^{N_x} \sum_{y=1}^{N_y} \begin{cases} 1, \text{if } I(x,y) = i \text{ and } I(x+d,y) = j, \\ 0, \text{otherwise,} \end{cases} \qquad \text{(S1)}$$

where $I(x,y)$ is the intensity of a pixel with coordinates (x,y).

Morphometric analysis and morpho-functional parameters, determined in animals with PAH

Morphometric analysis of the myocardium in animals with PAH showed the following changes [S5]. After 2 and 4 weeks, the wall thickness of the pancreas was statistically significantly higher than the control (p<0.05). After 1 month, the thickness of the pancreas progressively decreased and had no significant differences by the 8th week of observation. At the same time, the transverse size of the cavity of the right ventricle (CRV) and the diameter of the trunk of the pulmonary artery (PA) significantly increased compared with the control group (p<0.05) (see Tables S4 and S5).

Table S4 – The dynamics of the thickness of the walls of the heart chambers in the MCT-induced PAH, Me [25th; 75th percentile]

Groups of experimental animals	RV, mm [25%;75%]	IVS + LV, mm [25%;75%]	RV / (IVS + LV), mm [25%;75%]
Control	0,9 [0,7;1,0]	5,0 [5,0;6,5]	0,156 [0,111;0,2]
2 weeks of PAH	1,4* [1,0;1,6]	6,0 [4,5;7,0]	0,235 [0,167;0,3]
4 weeks of PAH	1,7* [1,2;2,0]	6,0 [6,0;6,0]	0,283** [0,2;0,3]
6 weeks of PAH	1,2* [1,1;2,0]	6,0 [6,0;7,0]	0,238 [0,117;0,333]
8 weeks of PAH	1,15 (1,0;1,5)	5,2 (5,0;6,0)	0,208 (0,2;0,231)

Notes:
* – the presence of significant differences in the thickness of the pancreas according to the Mann-Whitney test in comparison with the control group (p<0.05)
** – the presence of significant differences in RV/(IVL+LV) according to the Mann-Whitney test in comparison with the control group (p<0.05)
RV – the wall thickness of the right ventricle, mm
IVS + LV – the sum of the thickness of the interventricular septum and the free wall of the left ventricle

RV / (IVS + LV) – the ratio of the wall thickness of the right ventricle to the sum of the thickness of the interventricular septum of the LV free wall.

Table S5 – Time dynamics of the transverse dimensions of the cavities of the right heart and pulmonary arteries during the development of PAH induced by monocrotaline, Me [25th; 75th percentile]

Groups of experimental animals	RVC, mm [25%;75%]	PPP, mm [25%;75%]	Trunk LA, mm [25%;75%]
Control	3,2 [3,2; 3,3]	3,07 [3,0; 3,1]	2,9 [2,8; 3,1]
8 weeks of PAH	3,76* [3,7; 3,8]	5,49* [5,4; 5,6]	3,37* [3,2; 3,8]

Notes:
* – the presence of significant differences in values according to the Mann-Whitney test in comparison with the control group ($p<0.05$)
RVC – transverse dimension of the cavity of the right ventricle
PPP – transverse dimension of the cavity of the right atrium
trunk LA – diameter of the trunk of the pulmonary artery

Morphometric analysis of pulmonary vessels revealed the following changes [S6]. The development of MCT-induced PAH was accompanied by a significant increase in the thickness of the intima of small pulmonary capillaries, starting from the 2nd week, and the thickness of the medial layer – by 8 weeks of the experiment (Tables S6).

Table S6 – Index of thickening of the intima and media of small pulmonary arterioles (up to 35 µm)

Groups of experimental animals	Intima			Media		
	ITI, % Me (25;75)	95% CI	P	MTI, % Me (25;75)	95% CI	P
**Control**	1,63 (1,08; 2,87)	4,54-6,93	$P_{1-2}<0{,}001$ $P_{1-3}<0{,}001$ $P_{1-4}<0{,}001$ $P_{1-5}<0{,}001$	41,68 (29,70; 49,71)	15,38-23,47	$P_{1-2}=0{,}55$ $P_{1-3}<0{,}001$ $P_{1-4}=0{,}44$ $P_{1-5}<0{,}001$
**2 weeks of PAH**	10,90 (7,43; 19,33)	9,82-19,22	$P_{2-3}=0{,}07$ $P_{2-4}<0{,}001$ $P_{2-5}=0{,}12$	36,10 (26,95; 53,35)	11,60-22,70	$P_{2-3}=0{,}001$ $P_{2-4}=0{,}35$ $P_{2-5}=0{,}001$
**8 weeks of PAH**	19,62 (11,60; 24,67)	27,43-42,99	$P_{3-4}<0{,}001$ $P_{3-5}=0{,}001$	55,98 (47,71; 66,32)	15,97-25,03	$P_{3-4}<0{,}001$ $P_{3-5}=0{,}08$

Notes:
MTI – Media thickening index
ITI – Intima thickening index

We did not measure pulmonary artery pressure invasively. Hemodynamic parameters were assessed using an echocardiographic study (EchoCG) of the rat heart by indirect parameters, taking into account the technical difficulties in the determination of the hemodynamic parameters of the right heart due to the high heart rate of rats. The study was performed on an iE33 device (Philips Ultrasound, Bothell WA, USA) using a 12 MHz transducer according to generally accepted methods in M mode (one-dimensional echocardiography) and B mode (two-dimensional echocardiography).

EchoCG assessment of the studied parameters recorded a decrease ($p<0.05$) of the thickness of the interventricular septum, the posterior wall of the left ventricle in systole after 8 weeks of the experiment compared with the control group. Along with this, a decrease in the ejection fraction, shortening fraction, stroke volume of the left ventricle ($p<0.05$) was recorded at the same time of the experiment (Table S7).

The transverse size of the right ventricle gradually increased during the observation ($p<0.05$), and the thickness of the interventricular septum in systole increased after 2 weeks and did not differ from the control group ($p<0.05$) after 8 weeks.

Table S7 – Morphological and functional parameters of rats assessed by transthoracic echocardiography in MCT-induced PAH, Me [25;75]

**EchoCG parameters**	**Groups of experimental animals**		
	1 (control, n=12)	2 (2 weeks of PAH, n=10)	3 (8 weeks of PAH, n=11)
**IVSd, cm**	0,3 (0,2; 0,3)	0,45 (0,4; 0,5)*	0,3 (0,2; 0,3)#
**IVSs, cm**	0,45 (0,4; 0,5)	0,45 (0,4; 0,5)	0,3 (0,3; 0,3)*#
**LViDd, cm**	1,35 (1,3;1,4)	1,2 (1,0;1,3)	1,2 (1,2;1,6)
**LViDs, cm**	0,85 (0,8; 0,9)	0,65 (0,6;0,8)	1,1 (1,0;1,3)#
**LVPWd, cm**	0,3 (0,3; 0,3)	0,3 (0,2; 0,3)	0,3 (0,2; 0,3)
**LVPWs, cm**	0,5 (0,4; 0,5)	0,45(0,4; 0,5)	0,4(0,3; 0,4)*#
**EDV Teich, mL**	4,5 (4,0; 5,0)	3,5 (2,0; 4,0)	3,7 (3,0; 7,0)
**ESV Teich, mL**	1,0 (1,0; 3,0)	1,5 (1,0; 2,0)	1,8 (1,8; 1,9)
**EF, %**	69 (66,0; 71,0)	67 (65,0; 72,0)	42 (36,0; 64,0)*#
**SV, mL**	3,0 (3,0; 4,0)	2,0 (2,0; 3,0)	2,0 (1,0; 2,0)*
**FS, %**	36,5 (65,0; 40,0)	37,5 (36,0; 38,0)	18,0 (14,0; 33,0)*#
**RV, cm**	0,4 (0,4; 0,5)	0,6 (0,6; 0,7)	0,9 (0,8; 1,0)*#

Notes:
1 – *$p<0.05$ when compared with control group 1;
2 – #$p<0.05$ when compared with group 2.
IVSd – interventricular septum diastolic
IVSs – interventricular septum sistolic
LViDd – left ventricular end-diastolic dimension
LViDs – left ventricular end-sistolic dimension
LVPWs – thickness of left ventricular posterior wall sistolic
LVPWd – thickness of left ventricular posterior wall diastolic
EDV Teich – end-diastolic volume
ESV Teich – end-sistolic volume
EF – ejection fraction
SV – stroke volume
FS – fractional shortening
RV – Anteroposterior dimension of right ventricular

Thus, the data of the morphological analysis and the results of the echocardiographic study obtained by us convincingly testify to the consistency of the 8-week monocrotaline model of PAH.

References

S1. van Zuijlen, P. P. *et al.* Morphometry of dermal collagen orientation by Fourier analysis is superior to multi-observer assessment. *J. Pathol.* **198**, 284–291 (2002).

S2. Otsu, N. A Tlreshold Selection Method from Gray-Level Histograms. 5.

S3. Fitzgibbon, A., Pilu, M. & Fisher, R. B. Direct least square fitting of ellipses. *IEEE Trans. Pattern Anal. Mach. Intell.* **21**, 476–480 (1999).

S4. Haralick, R. M., Shanmugam, K. & Dinstein, I. Textural Features for Image Classification. *IEEE Trans. Syst. Man Cybern.* **SMC-3**, 610–621 (1973).

S5. Adzericho, I. E., Jacevich, O. N., Vladimirskaja, T. E. & Mihnevich, D. L. Morphological features in dynamics of monocrotaline-induced pulmonary arterial hypertension in vivo. *Pathol. Physiol. Exp. Med.* (2020).

S6. Akushevich, I., Yashkin, A. P., Kravchenko, J. & Yashin, A. I. Analysis of Time Trends in Alzheimer's Disease and Related Dementias Using Partitioning Approach. *J. Alzheimers Dis.* **82**, 1277–1289 (2021).
